# Immunological Mechanisms behind Anti-PD-1/PD-L1 Immune Checkpoint Blockade: Intratumoral Reinvigoration or Systemic Induction?

**DOI:** 10.3390/biomedicines12040764

**Published:** 2024-03-29

**Authors:** Zhikun Guo, Jiangnan Yu, Zihan Chen, Shuxian Chen, Lei Wang

**Affiliations:** International Cancer Center, Shenzhen University Medical School, Shenzhen 518054, China; 2100243054@email.szu.edu.cn (Z.G.); jiangnanyu@szu.edu.cn (J.Y.); 2200243013@email.szu.edu.cn (Z.C.); 2200243033@email.szu.edu.cn (S.C.)

**Keywords:** immune checkpoint blockade, tumor microenvironment, cancer-immunity cycle, lymph node, peripheral blood

## Abstract

Anti-PD-1/PD-L1 immune checkpoint blockade (ICB) has been widely used to treat many types of cancer. It is well established that PD-L1 expressing cancer cells could directly inhibit the cytotoxicity of PD-1^+^ T cells via PD-L1-PD-1 interaction. However, histological quantification of intratumoral PD-L1 expression provides limited predictive value and PD-L1 negative patients could still benefit from ICB treatment. Therefore, the current major clinical challenges are low objective response rate and unclear immunological mechanisms behind responding vs. non-responding patients. Here, we review recent studies highlighting the importance of longitudinal pre- and post-ICB treatment on patients with various types of solid tumor to elucidate the mechanisms behind ICB treatment. On one hand, ICB induces changes in the tumor microenvironment by reinvigorating intratumoral PD-1^+^ exhausted T cells (“releasing the brakes”). On the other hand, ICB can also affect systemic antitumor immunity in the tumor-draining lymph node to induce priming/activation of cancer specific T cells, which is evident by T cell clonal expansion/replacement in peripheral blood. These studies reveal that ICB treatment not only acts on the tumor microenvironment (“battlefield”) but also acts on immune organs (“training camp”) of patients with solid tumors. A deeper understanding of the immunological mechanisms behind ICB treatment will pave the way for further improvements in clinical response.

## 1. Introduction

Programmed death/ligand 1 (PD-1/PD-L1) are immune checkpoint molecules expressed on immune cells to regulate the immune responses in humans [1]. Immune checkpoint molecules function as an immunosuppressive brake, preventing the over-activation and occurrence of autoimmune diseases. However, cancer cells can also express immune checkpoint molecules to escape the killing behavior of cytotoxicity T cells. Immune checkpoint blockades (ICB) are therapeutic antibodies which are used to release the brake by blocking the interaction between immune checkpoint molecules, thereby promoting the anti-tumor activity of cytotoxicity T cells [2,3,4]. ICBs have been proved to be effective in various types of cancer, including non-small-cell lung cancer (NSCLC), breast cancer, melanoma, renal cell cancer, colorectal cancer, hepatocellular carcinoma, gastric cancer, and urothelial carcinoma [5,6,7,8]. Currently, approved ICB drugs target four immune checkpoints: CTLA-4, PD-1, PD-L1, and LAG-3, and this review focuses on PD-1/PD-L1. Notable anti-PD-1 antibodies like pembrolizumab, nivolumab, cemiplimab, tislelizumab, toripalimab, and camrelizumab, along with anti-PD-L1 antibodies such as atezolizumab, avelumab, and durvalumab, have become integral components of clinical cancer treatment.

Although responding patients can achieve long-term remission, the majority of patients do not benefit from ICB treatment. Patients with cancers who receive PD-1 blockade have response rates ranging from 40% to 70%, such as melanoma [9,10,11] and Hodgkin’s lymphoma [12], while the response rates are only 10% to 25% in most other approved cancers [5,6,13]. Current histological quantification of intratumoral PD-L1 expression provides limited predictive value and PD-L1 negative patients could still benefit from ICB treatment [14]. In addition, there are some limiting factors in the detection of tumor mutational burden, such as difficult sampling and unclear threshold definition [15]. However, even if patients initially respond to ICB, drug resistance may develop over time, leading to disease progression. The phenomenon of initially responding to ICB therapy but eventually experiencing clinical progression is termed acquired resistance [16]. A clinical study of 655 advanced melanoma patients treated with pembrolizumab showed that approximately 25% of melanoma responders experienced disease progression at a 21-month follow-up [17]. Additionally, in a clinical study of NSCLC patients treated with neoadjuvant nivolumab, up to 40% of responders progressed during a 5-year follow-up [18]. Therefore, the low objective response rate and unclear immunological mechanisms behind responding vs. non-responding patients are the major clinical challenges.

T cell activation requires the coordinated interaction between T cells and antigen-presenting cells (APCs) which mainly consist of dendritic cells, macrophages and B cells. The activation process of T cells involves multiple steps, including antigen presentation, co-stimulatory signals, and cytokine stimulation. Optimal antigen stimulation and appropriate co-stimulatory signals are crucial factors ensuring the correct activation, proliferation, and differentiation of T cells in the immune response [19,20]. ICBs are expected to restore T cell activity in the tumor microenvironment by releasing the immunosuppressive brake from cancer cells. However, recent studies have shown that ICB not only induces the mechanism for reinvigorating intratumoral exhausted T cells but also acts on systemic immunity. Therefore, the immunological mechanisms behind responding vs. non-responding patients with solid tumor still remain to be explored.

In this review, we focus on recent studies highlighting the importance of longitudinal pre- and post-ICB treatment, specifically focusing on PD-1/PD-L1, on patients with various types of solid tumor to elucidate the immunological mechanisms behind ICB treatment.

## 2. ICB-Induced Intratumoral Changes

**Figure 1:**
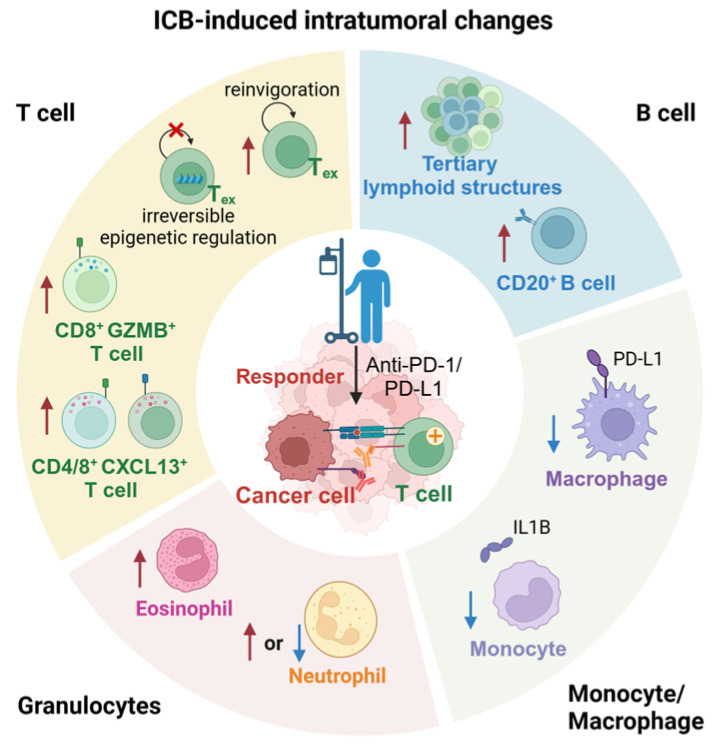
Immune checkpoint blockade induced intratumoral changes. Different background colors in the figure represent different types of immune cells. The arrows indicate the upregulation or downregulation of responder.

A tumor is a complex environment consisting of cancer cells, immune cells, and stromal cells [21]. Tumors were initially thought to be a disease caused by genetic mutations that cause cancer cells to overcome growth limitations and possess the ability to divide unlimitedly [22]. However, a small group of cancer cells does not have the ability to form tumors and metastasize. This complex tissue is often referred to as the tumor microenvironment (TME) [23], and every component of the TME has critical role in tumor therapy of ICB [24] (summarized in Figure 1).

### 2.1. Inactivation and Depletion of Regulatory T Cells

Cytotoxic T lymphocyte-associated protein 4 (CTLA-4) and PD-1 are both inhibitory immune checkpoint molecules expressed on T cells [8]. However, CTLA-4 is predominantly expressed on regulatory T cells, while PD-1 is expressed on antigen-specific activated T cells [20]. Although both anti-CTLA-4 and anti-PD-1 therapies can block the signaling pathways that inhibit T cell-mediated tumor killing, acting as a type of “release the brakes” mechanism for anti-tumor effects, their mechanisms of action differ. CTLA-4 primarily regulates the activation of APCs to T cells by competing with CD28 for binding to B7 [25]. On the other hand, PD-1 and PD-L1 mitigate T cell cytotoxicity against cancer cells, thereby alleviating the effector functions of T cells [26]. Anti-CTLA-4 therapy impacts the proliferation and trafficking of regulatory T cells (Treg), while anti-PD-1/PD-L1 therapy mainly affects CD8 T cells [27,28]. There have been other reviews to explain the mechanism of anti-CTLA-4 therapy [29,30,31,32]; therefore, this review aims to focus on anti-PD-1/PD-L1 therapy.

### 2.2. ICB-Induced Reinvigoration of Exhausted T Cells

T cells play a pivotal role in anti-tumor immunity, but persistent antigen stimulation results in the exhaustion of T cells [33]. Dysfunction of T cells within tumors is frequently associated with the accumulation of exhausted T cells, characterized by diminished effector activity despite their viability [33]. Wang et al. had collected tumors from patients with triple-negative breast cancer at three time points (baseline, early on-treatment, and post-treatment) [34]. The combination of anti-PD-L1 immunotherapy and chemotherapy can elevate the ratio of CD8^+^PD-1^+^ exhausted T cells interacting with cancer cells, although ICB is not the sole influencing factor [34]. Nevertheless, when considering the overall TME cell proportions, ICB can induce higher levels of CD8^+^PD-1^+^ exhausted T cells compared to chemotherapy alone. Yost et al. analyzed the single-cell RNA-sequencing (scRNA-seq) and T cell receptor-sequencing (TCR-seq) data from 11 patients with advanced basal cell carcinoma before and after anti-PD-1 immunotherapy in primary tumor [35]. They observed that the cell counts of activated and exhausted T cells increased post-treatment, supporting that ICB primarily influence the CD8^+^ T cells [35]. Following the ICB treatment, exhausted T cells undergo clonal expansion and express signature genes associated with T cell dysfunction, such as *HAVCR2* and *TIGIT* [35]. Recent analyses of head and neck squamous cell carcinoma [36] and breast cancer [37] have also revealed that responders to anti-PD-1 therapy exhibit higher expression of cytotoxicity genes in exhausted CD8^+^ T cells compared to non-responders.

### 2.3. Terminally Exhausted T Cells Due to Irreversible Epigenetic Regulation

Clinical trials have previously demonstrated the effectiveness of reversing T cell exhaustion through the administration of anti-PD-1 inhibitors [6,33]. However, recent studies increasingly indicate substantial irreversible epigenetic alterations in exhausted T cells, implying that their reinvigoration is not easily achievable [38,39]. Rather than reversing the exhaustion state of T cells, the role of ICB is more likely to impede the transition of T cells to an exhausted phenotype during the differentiation process within the tumor microenvironment [40]. A recent study revealed that PD-1 blockade could prevent precursor-like T cells from differentiating into terminally exhausted T cells in NSCLC [41].

### 2.4. ICB-Induced CD8^+^GZMB^+^ T Cells Expansion

According to the cancer-immunity cycle principle, the functions of T cells in anti-tumor immune responses encompass priming and activation of T cells in lymph nodes, as well as their infiltration into the TME for the purpose of eliminating cancer cells [42]. By utilizing three-dimensional imaging mass cytometry to analyze tumor tissue biopsies from breast cancer patients undergoing chemotherapy or a combination of chemotherapy and anti-PD-L1 immunotherapy at three different time points, Wang et al. revealed that the cell density and proportion of CD8^+^GZMB^+^ T cells interacting with cancer cells in the TME of patients responding to ICB treatment are significantly higher compared to non-responding patients [34]. Furthermore, patients receiving ICB treatment exhibit a higher proportion of CD8^+^GZMB^+^ T cell infiltration than those undergoing chemotherapy alone. By comparing the differences before and after PD-1 blockade, the study found that endothelial cells, fibroblasts, T cells, innate lymphocytes, and NK cells in colorectal cancer were significantly increased in patients with pathological complete response (pCR) but not in the non-pCR patients [43]. Moreover, they reveal that the CD8^+^ effector memory T cells have expressed granzymes and increased genes associated with antigen presentation and IFN-γ. These studies have all confirmed that ICB treatment can increase the proportion of CD8^+^ T cells and exhausted T cells.

### 2.5. ICB-Induced CXCL13^+^ T Cells Expansion

By analyzing the scRNA-seq and TCR-seq data from five kinds of cancer, including NSCLC, basal cell carcinoma, breast cancer, renal cell cancer and squamous cell carcinoma, Liu et al. revealed that the CD8^+^CXCL13^+^ T cells had correlated with response to ICB and the counts of these cells increased after ICB [41]. Treatment categories for the 102 analyzed patients are divided into the renal cell cancer patients, treated with anti-PD-1 or combination therapies of anti-PD-1 with anti-CTLA-4; and the other kinds of cancer patients, treated with anti-PD-1/PD-L1 or combination therapies of anti-PD-1/PD-L1 with chemotherapy. These results suggest that CD8^+^CXCL13^+^ T cells play an important role in the therapeutic process of ICB, and the degree of CD8^+^CXCL13^+^ T cells infiltration before treatment can predict the efficacy of ICB. Due to the modulatory function of CD4^+^ T cells on CD8^+^ T cells, they explored the changes in CD4^+^ T cells after ICB and found that the counts of CD4^+^CXCL13^+^ T cells after treatment in responding patients were higher than in non-responding patients. Moreover, they found that responders had a high abundance of both CD8^+^CXCL13^+^ T cells and CD4^+^CXCL13^+^ T cells compared to non-responders. This suggests that the proportion of CXCL13^+^ T cells to total T cells could be a marker for predicting ICB response. Meanwhile, Cohen et al. analyzed the scRNA-seq data obtained from 36 patients with breast cancer; then, they also revealed that the counts of CD4^+^PD-1^+^CXCL13^+^ T cells had significantly increased after receiving anti-PD-1 therapy [44].

### 2.6. ICB-Induced CD20^+^ B Cells Expansion

Although ICB treatment primarily focuses on T cells, an increasing body of research data has revealed the crucial function of tumor-associated B cells (TABs) [45]. TABs can modulate the immune response and shape the cellular composition of the tumor microenvironment by releasing chemokines, including regulatory T cells and macrophages [46]. By using deconvolution to perform cell type enrichment scores in bulk RNA-sequencing to compare the abundance of B cells in tumor tissue and paired normal tissue, Laumont et al. found that in most immune “hot” tumors, tumor tissue had significantly more B cells than normal tissue [45] and it could help to build tertiary lymphoid structures (TLSs), which are new lymph node-like structures promoting immune response [45]. B cells are produced and mature in the bone marrow, where they undergo V(D)J recombination, leading to cell surface expression of IgM isotype B cell receptors [47]. Upon encountering pathogens, B cells can recognize, memorize, and secrete antibodies to eliminate pathogens. The advent of flow cytometry and single-cell RNA-sequencing has provided a more comprehensive understanding of B cell subtypes, with more than 10 reported subpopulations of B cells identified in human blood [48], lymphoid tissue [49,50], and peripheral tissue [51].

In the studies of human cancer, TABs encompass naive, activated, and memory B cells, germinal center B cells, and plasma cells and their intermediates [52]. scRNA-seq also contributes to an increased understanding of TAB phenotypes, which is beginning to inform prognostic studies. For example, tumor tissue from nasopharyngeal carcinoma identify that IgD^−^CD27^−^ B cells and plasma cells are negatively and positively correlated with survival rate, respectively [53]. Conversely, scRNA-seq data of tumor tissue from colorectal cancer show that the IgA^+^IGLC2^+^ plasma cells subset is associated with poor prognosis [54]. Wang et al. reveal that the counts of B cells show a declining trend in responders to ICB, although the proportions of B cells have remained stable [34]. By using the multiplex immunostaining and scRNA-seq, Griss et al. observed that anti-CD20 therapy induces plasmablast-like B cells, a distinct subtype of B cells, with downregulated expression of TGFB1 and IL-10 [46]. Li et al. prove that the proportion of CD20^+^ B cells has increased in pCR patients who received anti-PD-1 therapy compared with non-pCR patients. They found that the high abundance of CD20^+^ B cells in tumors was accompanied by a high abundance of CD8^+^ effector memory T cells and low abundance of CD8^+^ tissue resident memory mitotic cells [43]. This suggests that CD20^+^ B cells can regulate the status of CD8^+^ T cells and can promote the response to ICB [43]. The functional role of TABs in patients’ anti-tumor immunity remains unclear. B cells are highly enriched in tertiary lymphoid structures, influencing patients’ responses to ICB. Research suggests that B cells secrete antibodies and coat tumor cells, contributing to anti-tumor immune responses in ovarian cancer [55,56] and lung cancer [57]. Additionally, B cells expressing PD-1 and PD-L1 can impact immune therapy through the PD-1/PD-L1 pathway. TABs show great diversity in subtypes and heterogeneity in different tumors, and require more research to discover the connection between TABs and ICB clinical outcomes.

### 2.7. Abundant Tertiary Lymphoid Structures in ICB Responding Patients

Tertiary lymphoid structures (TLSs) are lymph node-like structures containing a B cell zone in contact with an adjacent T cell zone [58]. The abundance of B cells within the TME is highly dependent on the maturity of TLSs [58,59]. To comprehensively understand the distribution of B cells in the tumor tissue of ICB responders and their relationship with TLSs, researchers conducted histological assessments of tumor samples obtained from melanoma patients [60]. The results indicated that the expression of CD20, the density of TLSs, and the ratio of TLSs to tumor area were higher in responder tumor tissues compared to non-responders, especially in samples from early-stage treatments. Further spatial analysis revealed that CD20^+^ B cells were localized within the TLSs of responder tumors, co-localizing with CD4, CD8, and FOXP3 T cells. Another study, through pathological analysis of slices from patients undergoing ICB treatment, found a positive correlation between mature TLSs, characterized by the presence of CD23^+^ follicular dendritic cells, and favorable prognosis [61].

### 2.8. ICB-Induced Tumor-Associated Macrophages Reprograming

Myeloid cells, primarily derived from bone marrow and spleen, constitute a crucial component of the immune system, including monocytes/macrophages, dendritic cells, myeloid-derived suppressor cells (MDSCs), and granulocytes [62]. Tumor-associated macrophages (TAMs) are the most abundant immune cells in the tumor microenvironment [63], dendritic cells possess antigen-presenting functions activating adaptive immunity, MDSCs are associated with distant tumor metastasis [64], and neutrophils contribute to extracellular matrix remodeling with both pro-inflammatory and immune-suppressive effects [65]. Myeloid cells play a significant role in killing tumor cells during tumor development, activating T cells through co-stimulatory signals, and exerting anti-tumor effects [66]. Studies have shown that tumors can remotely activate the differentiation and immune phenotype of myeloid lineage cells, including MDSCs, in the bone marrow or the spleen through cytokines, recruiting more tumor-promoting myeloid cells [67]. These findings suggest that the development and progression of tumors can influence the differentiation of myeloid lineage cells in the bone marrow and spleen, as well as the generation and migration of tumor-infiltrating myeloid cells. Recent research indicates that ICB treatment also induces immune changes in tumor-associated macrophages.

After breast cancer patients were treated with anti-PD-1, the macrophage phenotypes expressing PD-L1, including CCR2^+^ and MMP9^+^ macrophages, correlated positively with T cell expansion [37]. By contrast, naive or effector/memory T cells, and inhibitory (CX3CR1^+^) macrophages, were inversely correlated [37]. By studying colorectal cancer patients who received neoadjuvant PD-1 blockade and performing scRNA-seq on their tumor and adjacent normal tissue samples, Li et al. found that myeloid cell subsets were reshaped after ICB treatment, with a significant reduction in the proportion of IL1B^+^ monocytes cells in pCR patients [43]. IL1B^+^ monocytes highly expressed the proinflammatory factors and had tumor-promoting function by regulation of the epithelial–mesenchymal transformation pathway. By contrast, the non-pCR patients had more proinflammatory IL1B^+^ monocytes, suggesting that the failure of inflammation resolution may lead to the non-response of ICB. These studies collectively suggest that ICB treatment influences the immune phenotype of tumor-infiltrating myeloid cells.

### 2.9. ICB-Induced Neutrophils Expansion in Non-Responders

By analyzing bulk RNA sequencing results from tumors collected from triple-negative breast cancer patients before and after anti-PD-1 therapy, Blomberg et al. found that eosinophils in response patients were significantly upregulated after treatment, and the eosinophils could promote the activation of CD8^+^ T cells [68]. Hirschhorn et al. collected the biopsies from melanoma patients before and after treatment with anti-CTLA-4 and/or anti-PD-1 therapy [69]. They found that the activation of neutrophils had increased after ICB, as detected by the formation of extracellular traps staining. On the contrary, another study showed that 62 CRC patients with poor response to PD-1 blockade had increased neutrophil infiltration, and a high neutrophil-to-lymphocyte ratio could be a predictor of a poor response to ICB [70].

## 3. ICB-Induced Changes in Peripheral Blood T Cells

**Figure 2:**
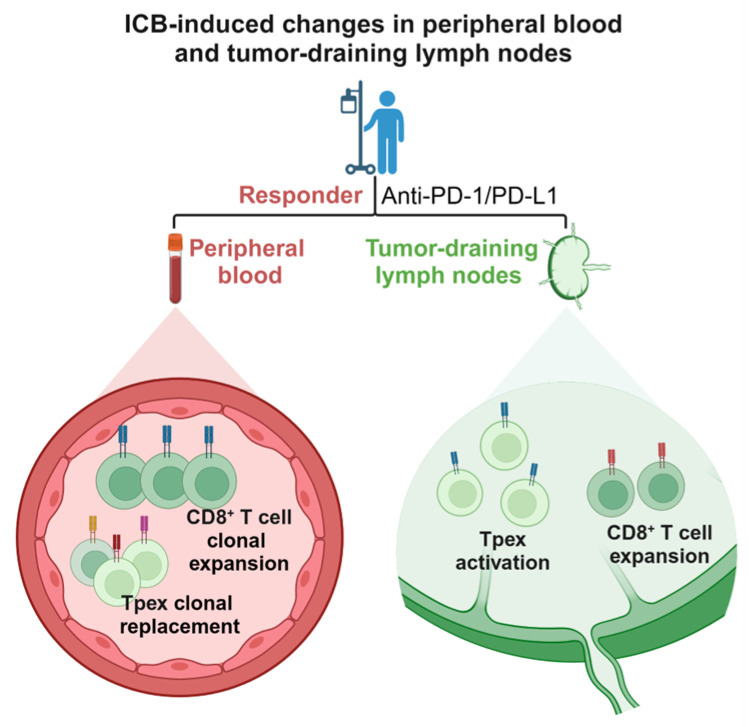
Immune checkpoint blockade induced changes in peripheral blood and tumor-draining lymph nodes. Figures created with BioRender.com (accessed on 25 March 2024).

Peripheral blood plays a crucial role in anti-tumor immunity, serving as a bridge between tumors and lymph nodes [71]. It comprises various cell types, including red blood cells, platelets, and immune cells. Peripheral blood has the advantages of convenient sampling and minimal invasiveness compared to tumor tissue biopsy. Monitoring the change of biomarkers in peripheral blood is a superior strategy for predicting the response to ICB [72]. Therefore, the alterations in peripheral blood cells of patients undergoing ICB treatment are also noteworthy (summarized in Figure 2).

### 3.1. ICB-Induced Clonal Replacement of T Cells in Peripheral Blood

Yost et al. proposed the concept of “clonal replacement” and found that in the immunotherapy of lung cancer, both novel T cell clones and pre-existing T cell clones can be recruited to the tumor to exert their functions [35]. They suggest that the infiltration of new clonotypes (clonal replacement) after ICB symbolizes vital changes in intratumoral T cells. Liu et al. observed an increased proportion of progenitors of exhausted T (Tpex) in the tumors of lung cancer patients responding to immunotherapy [73]. Importantly, this rise in Tpex cells was not attributed to the reversal of terminally exhausted T cells; rather, it resulted from the expansion of pre-existing exhausted T cell clones and the supplementation of new T cell clonotypes from peripheral blood before treatment. These findings reveal that ICB treatment induces not only clonal expansion of T cells within the tumor but also the emergence of new clonotypes in the peripheral blood [73]. Furthermore, these studies collectively suggest that a systemic anti-tumor immune response requires the generation of new T cell clones outside the tumor by promoting tumor immune circulation.

### 3.2. ICB-Induced Clonal Expansion of T Cells in Peripheral Blood

Fairfax et al. analyzed single-cell transcriptome sequencing and TCR-seq data from peripheral blood mononuclear cells from 55 melanoma patients before and after anti-PD-1 alone or in combination with anti-CTLA-4. They suggest that the responding patients have more CD8^+^ T cell clones post-treatment than the non-responding patients [74]. Anti-PD-1 combination with anti-CTLA-4 has more significant therapeutic efficacy than only anti-PD-1 treatment. Moreover, there are more large clones, occupying >0.5% in the repertoire in the responders, and the gene overexpression of large clones is related to the effector memory T cell such as *CCL4*, *NKG7*, and *GNLY*. These suggest that the change of CD8^+^ T cell in peripheral blood can reveal the treatment response of ICB. Wu et al. conducted mRNA and TCR sequencing on tumors, adjacent normal tissues, and peripheral blood of patients across four cancer types, including lung cancer, endometrial adenocarcinoma, colorectal cancer, and renal cell cancer, revealing dynamic changes in T cells post-immunotherapy [75]. Notably, there was an expansion of entirely new T cell clones in tumors, adjacent normal tissues, and peripheral blood, correlating with improved immune therapy response. These findings suggest that intratumoral T cells in responsive patients may originate from the generation of new, non-exhausted T cells outside the tumor. Alterations in non-exhausted T cell clonotypes in peripheral blood can serve as predictive indicators of patient responsiveness to immunotherapy [75].

## 4. ICB-Induced Changes in Tumor-Draining Lymph Nodes (TDLNs)

### 4.1. Cancer-Immunity Cycle

In order to better understand the mechanism by which the immune system eliminates tumors, Chen et al. proposed the concept of the cancer-immunity cycle in 2013 [42]. Initially, cancer cells, upon destruction, release antigens that are captured and processed by APCs. Subsequently, these APCs enter the lymph nodes through the peripheral blood, presenting the captured antigens on major histocompatibility complex (MHC) molecules to T cells. Activated T cells within the lymph nodes then infiltrate the tumor through the peripheral blood, executing the destruction of cancer cells. The killed cancer cells release more tumor-associated antigens, deepening and broadening the tumor immune cycle. According to the theory of cancer-immunity cycle, generating an anti-tumor immune response requires changes not only within the tumor microenvironment but also in the immune macroenvironment outside the tumor.

According to the cancer-immunity cycle theory, tumor-draining lymph nodes (TDLNs) play a critical role in anti-tumor immune surveillance and response. Previous studies have indicated that the anti-tumor immune response in the tumor microenvironment is closely linked to the immune macroenvironment outside of the tumor, with TDLNs serving as a crucial site for de novo generation and activation of frontline anti-tumor T cells. During the process of tumor initiation and progression, a reprogramming of the microenvironment within lymph nodes occurs, leading to the remodeling of the stroma in TDLNs [76,77]. This results in the formation of lymphatic vessels, the expansion of high endothelial venules, and the establishment of an early metastatic niche.

### 4.2. ICB-Induced Tpex Activation in TDLNs

Additionally, TDLNs serve as crucial sites for the generation and activation of de novo anti-tumor T cells. Progenitors of exhausted T (Tpex) cells, identified as precursor cells of CD8^+^ exhausted T cells, manifest features of depletion and memory in the context of chronic infections and tumors [78].

Huang et al. reported the presence of tumor antigen-specific memory CD8^+^ T cells in TDLNs of mice [79]. A subset of these immune cells exhibited a PD-1^+^TCF-1^+^TOX^−^ phenotype reminiscent of classical immune memory cells, playing a crucial role in the response to ICB treatment. The specific CD8^+^ T cell subset in TDLNs can further differentiate and infiltrate into the tumor, continuously replenishing the exhausted T cells in the tumor tissue [79]. Simultaneously, Prokhnevska et al. discovered that in prostate and kidney cancers, activated CD8^+^ T cells in the TDLNs exhibited similarities in transcriptional levels and functionality with TCF1^+^ stem-like cells within the tumor [19]. Rahim et al. employed single-cell genomics and mass cytometry to interrogate CD8^+^ T cells in human head and neck squamous cell carcinoma, TDLNs, and peripheral blood [80]. They observed a substantial presence of Tpex in TDLNs, establishing a clonal relationship with terminally exhausted CD8^+^ T cells in the tumor. They investigated uninvolved lymph nodes of patients undergoing anti-PD-L1 therapy. The results revealed that ICB influenced the levels of Tpex and intermediate-exhausted CD8^+^ T cells (Tex-int) in uninvolved lymph nodes. After ICB treatment, Tpex levels decreased in uninvolved lymph nodes, while Tex-int cells proliferated. Moreover, these proliferative Tex-int cells appeared near dendritic cells, indicating the activation and differentiation of Tpex.

Tumor-derived factors can influence the functions of immune and stromal cells within TDLNs, modulating their roles in anti-tumor immunity and thereby impacting the systemic anti-tumor immune response in patients. However, how TDLNs mobilize systemic anti-tumor immune responses and impact patients’ response to immune ICB treatment remains to be investigated. The evidence from these studies collectively indicates that activating a systemic anti-tumor immune response requires not only the restoration of the anti-tumor activity of exhausted T cells already infiltrated the tumor but also the activation and proliferation of T cells within lymph nodes. This process induces the initial activation of entirely new (de novo) anti-tumor T cells, which infiltrate the tumor via peripheral blood, thereby driving the generation of an immune response within the tumor (summarized in Figure 2).

## 5. Conclusions

In this review, we summarize the changes in the tumor microenvironment and the immune macroenvironment of patients before and after receiving ICB treatment. In the tumor microenvironment, PD-1 is not only expressed on T cells, but also on other immune cells such as B cells and NK cells. PD-L1 is also expressed on cancer cells, macrophages, B cells and other cells. Recent study has shown that PD-L1 on T cells interact with CD80 on APCs, thereby affecting the function of PD-1 on these T cells [81]. These indicate the complexity of ICB treatment and the immune microenvironment.

In the tumor microenvironment, ICBs block the co-inhibitory receptors of T cells, such as PD-1 and CTLA-4, and inhibit the inhibitory signals of antigen-specific T cells, enabling them to attack tumor cells more effectively. ICB therapy can activate and enhance the function of CD8^+^ T cells, making them more persistently active, which helps to form a stronger immune response. This involves the reinvigoration and expansion of exhausted T cells and the expansion of Tpex cells. In addition to T cells, various immune cells also contribute significantly to the anti-tumor immune response of ICBs, including the expansion of CD20^+^ B cells and the abundance of tertiary lymphoid structures. This local immune response helps to change the immune suppressive state of the tumor microenvironment and enhances the ability of the immune system to recognize and attack tumors.

By studying the changes in peripheral blood and tumor-draining lymph nodes, we found that ICB treatment produced significant effects in systemic immune macroenvironment. In peripheral blood, ICBs caused the clonal expansion of CD8^+^ T cells, and also induced the expansion of Tpex in peripheral blood and lymph nodes, as well as the emergence of new T cell clones. This indicates the widespread impact of ICBs on the systemic immune system, thereby promoting the systemic immune response against tumors. At the same time, the specific CD8^+^ T cells in the tumor-draining lymph nodes can further differentiate and migrate to the tumor tissue, compensating for the loss of exhausted CD8^+^ T cells in the tumor. Therefore, the changes in the tumor-draining lymph nodes provide important support for the anti-tumor immune response.

In summary, further understanding of the mechanisms behind the changes induced by ICB can contribute to improving clinical response rates for patients with solid tumors. However, the resistance of ICB is still an important challenge faced by the current research. In order to allow more patients to benefit from ICB, the mechanisms of acquired resistance need to be further explored. We believe that combined chemotherapy or targeting other immune checkpoints are possible ways to overcome its resistance.
